# Use of sildenafil in an infant with persistent pulmonary hypertension secondary to lung and renal hypoplasia – a case report

**DOI:** 10.1186/s12887-019-1801-3

**Published:** 2019-11-06

**Authors:** Karen Lavie-Nevo, Kevin C. Harris, Joseph Y. Ting

**Affiliations:** 1 Department of Pediatrics, Carmel Medical Center, Haifa, Israel; 2 Department of Pediatrics, University of British Columbia, 1N16-4480 Oak Street, Vancouver, BC V6H 3V4 Canada

**Keywords:** Pulmonary hypertension, Sildenafil, Lung hypoplasia, Renal hypoplasia, Targeted neonatal echocardiography

## Abstract

**Background:**

Premature preterm rupture of membranes (PPROM) is reported to be associated with high rates of neonatal mortality and morbidity. Sildenafil has been used in infants with persistent pulmonary hypertension of newborn (PPHN) due to congenital diaphragmatic hernia (CDH) and bronchopulmonary dysplasia (BPD). Recently, Sildenafil has been evaluated as an alternative or adjunctive pulmonary vasodilator. This case report illustrates the use of early sildenafil for PPHN and right ventricular dysfunction in an unusual setting of lung and renal hypoplasia.

**Case presentation:**

A male infant was born at 37 weeks with a birth weight of 2840 g. Rupture of membranes developed at approximately 24 weeks of gestational age (GA). Bilateral small kidneys (< 2 standard deviations below average) were detected on ultrasound (US) examination at 30 weeks of gestation. The baby developed pneumothorax and pulmonary hypertensive crisis towards the end of the first day. An echocardiogram showed a dilated right ventricle, moderate right ventricular systolic dysfunction, hypoplastic pulmonary arteries and a large patent ductus arteriosus with bidirectional flow. The patient was sedated, paralyzed, and inhaled nitric oxide was administered to decrease the pulmonary resistance. In anticipation of persistent pulmonary hypertension due to the hypoplastic lungs and small calibre of pulmonary arteries, sildenafil was started on day of life (DOL) 5 at a dosage of 0.25 mg/kg/dose Q8H and gradually increased to 2 mg/kg/dose Q8H on DOL 9. The patient was finally extubated on DOL 7 and weaned off of non-invasive respiratory support on DOL 26. Sildenafil was gradually weaned beginning on DOL 21 and discontinued on DOL 48. Repeat echocardiogram assessment at 3 months showed complete resolution of PHT and right ventricular dilatation.

**Conclusions:**

We describe the early use of sildenafil in treating pulmonary hypertension associated with lung and renal hypoplasia in a non-CDH patient. Following this treatment the patient made a full recovery from right ventricular dysfunction.

## Background

Mid-trimester PPROM with prolonged oligohydramnios remains a challenge for both obstetricians and neonatologists. Although survival rates have improved, morbidity remains common particularly due to pulmonary insufficiency and pulmonary hypertension [1]. Oligohydramnios due to second trimester PPROM or impaired foetal kidney development may cause pulmonary hypoplasia [2]. These newborns present with severe respiratory failure secondary to airway and vascular development abnormalities resulting in alveolar hypoplasia, pulmonary artery musculature hyperplasia, ventilation perfusion mismatch and often persistent pulmonary hypertension (PPHN) which may be partially reversible [3]. Contemporary neonatal intensive care strategies like the use of volume-targeted and high frequency ventilation, combined with early treatment of PPHN with inhaled nitric oxide (iNO), have contributed to recent improvement in outcomes. Sildenafil has been used in infants with PPHN due to congenital diaphragmatic hernia (CDH) and bronchopulmonary dysplasia (BPD) [4]. Recently, Sildenafil has been evaluated as an alternative or adjunctive pulmonary vasodilator in PPHN [5]. A Cochrane Data Base Systematic Review published in 2017 states that the therapeutic mainstay for PPHN consists of assisted ventilation and administration of iNO, however iNO is costly and approximately 30% of the patients fail to respond to iNO [6]. High concentrations of phosphodiesterases in the pulmonary vasculature have led to the use of phosphodiesterases inhibitors such as sildenafil. We describe a term infant with lung hypoplasia secondary to both mid-trimester PPROM and renal hypoplasia complicated by PPHN and right heart dysfunction. The infant was treated with iNO and a two-month course of sildenafil. The right heart function normalized before hospital discharge.

## Case presentation

A male infant was born at 37 weeks with a birth weight of 2840 g. Rupture of the membranes developed at approximately 24 weeks of gestational age (GA), and serial ultrasounds (US) revealed progressively worsening oligohydramnios. Bilateral small kidneys (< 2 standard deviations below average) were detected on US examination at 30 weeks of gestation. Antenatal dexamethasone was given and the infant was delivered by caesarean section at 37 weeks GA due to severe oligohydramnios. He received intermittent positive pressure ventilation for a brief period and was then administered continuous positive airway pressure (CPAP). His Apgar scores were 5 at 1 min and 7 at 5 min. The infant developed right pneumothorax requiring chest needle compression shortly after admission into the neonatal intensive care unit (NICU). Empirical antibiotics were given followed by a negative culture. Towards the end of first day of life, there was re-accumulation of the right sided pneumothorax; a chest drain was inserted, and the patient was intubated and placed on a high frequency jet ventilator. He then developed multiple episodes of profound desaturations, increased pre- and post-ductal saturation differences (> 20%) and hypotension compatible with pulmonary hypertensive crisis. An echocardiogram showed a dilated right ventricle, moderate right ventricular systolic dysfunction, hypoplastic pulmonary arteries (2.8 mm bilaterally) and a large patent ductus arteriosus with bidirectional flow (Table 1). The patient was sedated, paralyzed, and inhaled nitric oxide was administered at 20 ppm to decrease the pulmonary resistance. FiO2 dropped from 100 to 21%, and oxygenation index decreased from 37 to 3.9 within 3 h. Low dose epinephrine at 0.05 micrograms/kg/min and alprostadil infusion at 0.005 μg/kg/min were added. Hydrocortisone was administered (up to 1 mg/kg/dose Q8H) for 4 days. In anticipation of persistent pulmonary hypertension due to the hypoplastic lungs and small calibre of pulmonary arteries, sildenafil was started on day of life (DOL) 5 at a dosage of 0.25 mg/kg/dose Q8H and gradually increased to 2 mg/kg/dose Q8H on DOL 9. Alprostadil infusion and iNO were weaned off on DOL 6 and DOL 7, respectively. The patient was finally extubated on DOL 7 and weaned off of non-invasive respiratory support on DOL 26. Despite the elevated serum creatinine (159umol/L on DOL 4), there were no significant fluid or electrolyte disturbances necessitating renal replacement therapy. Serial targeted neonatal echocardiograms (TNE) showed gradual improvement of the PPHN with time (Table 1). Sildenafil was gradually weaned beginning on DOL 21 and discontinued on DOL 48 (2 days before discharge). Repeat echocardiogram assessment at 3 months showed complete resolution of PHT and right ventricular dilatation. The infant is regularly followed up for growth and chronic renal disease.

**Table 1:** Serial targeted neonatal echocardiography findings of the indexed baby

Echocardiogram findings					
Days of life	Day 2	Day 3	Day 5	Day 8	Day 48
RV dilatation	Moderate	Moderate	Mild	Mild	No
RV FAC (%)	29	57	42	52	58
TAPSE (cm)	0.7	0.7	0.85	0.9	N/A
PDA direction	Bidirectional L➔ R 70%	R➔L	Bidirectional L➔ R 70%	L➔R	closed
LV FS (%)	34	29	48	47	N/A^a^
LVO (ml/kg/min)	70	74	151	255	287
Interventions (at the time of echo)					
NO	–	+	+	–	–
PGE	–	–	+	–	–
Sildenafil	–	–	+	+	+

*N/A* Not available, *RV* Right ventricle, *RVFAC* Right ventricle fractional area change, *TAPSE* Tricuspid annular plane systolic excursion, *PDA* Patent ductus arteriosus, *LV FS*, Left ventricle fractional shortening, *LVO* Left ventricular output, *NO* Nitric oxide, *PGE*, Prostaglandin, *LVEF* Left ventricle ejection fraction, *DOL* Day of life

^a^N/A: not available; quantitative measurement of LVEF was not performed due to movement artefacts, although LV contractility was unremarkable on eye balling

## Discussion and conclusions

The patient described in this report had PPROM before 25 weeks (prior to the saccular stage of lung development) with severe oligohydramnios and renal hypoplasia. He developed hypoxic respiratory failure and right ventricular dysfunction, secondary to lung hypoplasia and PPHN, and sildenafil was started to facilitate weaning of iNO. This report illustrated the use of early sildenafil for PPHN and right ventricular dysfunction in an unusual setting of lung hypoplasia unrelated to CDH condition (PPROM and renal hypoplasia).

Pulmonary hypoplasia secondary to second trimester PPROM and oligohydramnios presents a challenge to neonatal caregivers, although survival to discharge has improved substantially up to 70% in large series [2]. The pressure of the foetal lung fluid is essential for growth and morphogenesis of the developing lung, as normal transition to the saccular stage with the development of blood-gas interface requires the production of fluid by the foetal lung. Increased arteriolar muscularization, abnormal capillary formation, and thickening of the blood-gas barrier were demonstrated in autopsy samples from neonates with PPROM > 14 days [7].

Most existing literature focuses on PPHN associated with pulmonary hypoplasia in the context of CDH [8–10]. NO is produced from the nitrogen of L-arginine and molecular oxygen by the endothelial nitric oxide synthase enzyme (eNOS) in vascular endothelial cells. NO diffuses through the endothelium to the vascular smooth muscle and activates soluble guanylyl cycles (sCG) leading to the production of cyclic guanyl monophosphate (cGMP) which activates the cGMP-dependent protein kinase or protein kinase G (PKG). PKG activates potassium channels and reduction of the influx of calcium which leads to vasodilatation [11, 12]. Thus, iNO induces pulmonary vasodilatation by increasing intracellular cyclic guanosine monophosphate (cGMP) concentrations [4]. Sildenafil is a potent and highly selective inhibitor of phosphodiesterase type 5 (PDE5) that reduces the degradation of cGMP, resulting in nitric oxide mediated vasodilatation [3]. It can be administered orally or intravenously. Its off-label use in term and premature infants with PPHN is increasing, with limited safety or efficacy data [13]. Currently, sildenafil is used for the following indications in neonates: (a) as an acute adjuvant to iNO in iNO-resistant PPHN or to facilitate weaning from iNO; (b) as an acute primary treatment of PPHN when iNO is not available or is contraindicated and (c) in the chronic primary treatment of pulmonary hypertension for conditions such as BPD and CDH to decrease right ventricular pressures and potentially improves right ventricular function over time [4].

In conclusion, we describe a single case with the early use of sildenafil to treat PPHN in a non-CDH patient, secondary to PPROM and lung & renal hypoplasia. Following this treatment, the patient made a full recovery. Further evidence is required to determine whether this treatment could be viable for all patients who present similar type of condition.

## Data Availability

The database used and analysed during the current case report are available from the corresponding author on reasonable request.

## Abbreviations

BPD: Bronchopulmonary dysplasia
CDH: Congenital diaphragmatic hernia
cGMP: Cyclic guanyl monophosphate
CPAP: Continuous positive airway pressure
DOL: Day of life
eNOS: Endothelial nitric oxide synthase enzyme
FAC: Fractional area change
FS: Fractional shortening
GA: Gestational age
iNO: Inhaled nitric oxide
LV: Left ventricle
LVO: Left ventricular output
NICU: Neonatal intensive care unit
NO: Nitric oxide
PDE5: Phosphodiesterase type 5
PGE: Prostaglandin
PKG: Protein kinase G
PPHN: Persistent pulmonary hypertension
PPROM: Preterm premature rupture of membrane
RV: Right ventricle
sCG: Soluble guanylyl cycles
TAPSE: Tricuspid annular plane systolic excursion
TNE: Targeted neonatal echocardiography
US: Ultrasound

## References

[1] Williams O, Hutchings G, Hubinont C (2012). Pulmonary effects of prolonged Oligohydramnios following mid-trimester rupture of membranes – antenatal and postnatal management. Neonatology.

[2] Everest NJ, Jacobs SE, Davis PG, Begg L, Rogerson S (2008). Outcomes following prolonged preterm premature rupture of the membranes. Arch Dis Child Fetal Neonatal Ed.

[3] Shah DM, Kluckow M (2011). Early functional echocardiogram and inhaled nitric oxide: usefulness in managing neonates born following extreme preterm premature rupture of membranes (PPROM). J Paediatr Child Health.

[4] Lakshminrusimha S, Mathew B, Leach CL (2016). Pharmacologic strategies in neonatal pulmonary hypertension other than nitric oxide. Semin Perinatol.

[5] Al Omar S, Salama H, Al HM (2016). Effects of early adjunctive use of oral sildenafil and inhaled nitric oxide on the outcome of pulmonary hypertension in newborn infants. A feasibility study. J Neonatal-Perinatal Med.

[6] Kelly LE, Ohlsson A, Shah PS. Sildenafil for pulmonary hypertension in neonates. Cochrane Database Syst Rev. 2017;8:CD005494. 10.1002/14651858.CD005494.pub4 PMC6483280 28777888

[7] Geary C, Whitsett J (2002). Inhaled nitric oxide for oligohydramnios-induced pulmonary hypoplasia: a report of two cases and review of the literature. J Perinatol.

[8] Chandrasekharan PK, Rawat M, Madappa R (2017). Congenital Diaphragmatic hernia-a review. Matern Health Neonatol Pernatol.

[9] Gien J, Kinsella JP (2016). Management of pulmonary hypertension in infants with congenital diaphragmatic hernia. J Perinatol.

[10] Kumar VHS, Dadiz R, Koumoundouros J (2018). Response to pulmonary vasodilators in infants with congenital diaphragmatic hernia. Pediatr Surg Int.

[11] Pedersen Jonas, Hedegaard Elise R., Simonsen Ulf, Krüger Marcus, Infanger Manfred, Grimm Daniela (2018). Current and Future Treatments for Persistent Pulmonary Hypertension in the Newborn. Basic & Clinical Pharmacology & Toxicology.

[12] Lai MY, Chu SM, Lakshminrusimha S (2018). Beyond the inhaled nitric oxide in persistent pulmonary hypertension of the newborn. Pediatr Neonatol.

[13] Perez KM, Laughon M (2015). Sildenafil in term and premature infants: a systematic review. Clin Ther.

